# Supplementary material for: Identification and Validation of Toxoplasma gondii Mitoribosomal Large Subunit Components
Source: Microorganisms. 2022 Apr 21;10(5):863. doi: 10.3390/microorganisms10050863 (PMC9145746; doi:10.3390/microorganisms10050863)
Supplement: Supplementary file 1 [file microorganisms-10-00863-s001.zip › Figure S3.pdf]

(**A**)

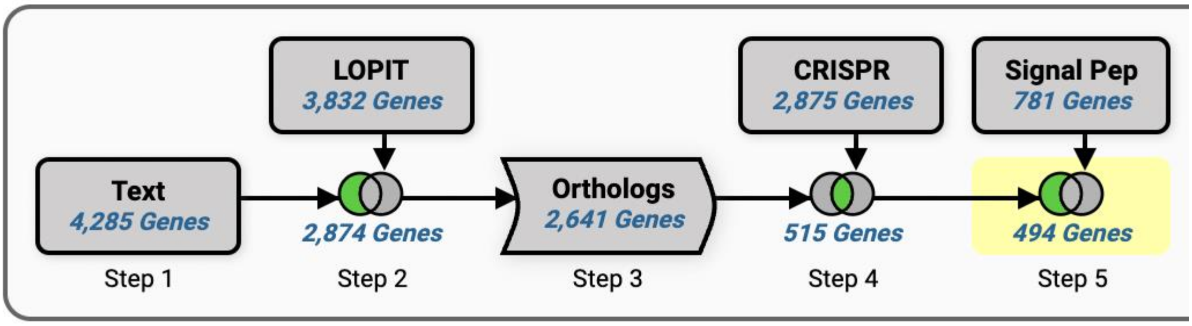

(**B**)

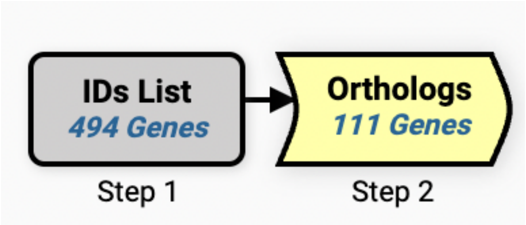

Figure S3. – Strategy captures from ToxoDB.org, showing how were the candidates in Table S3 found. (**A**) Step 1 – we selected all Toxoplasma gondii ME49 genes that have "hypothetical" in their product description, removed all genes for which there is a predicted localization. These genes were converted to their GT1 orthologs and then we selected all genes with fitness scores between -2 and -6.7 and removed those whose product has a predicted signal peptide. (**B**) Step 2 – we converted the 494 genes from step 1 to their orthologs from Plasmodium falciparum 3D7 ,which results in the 111 genes found in Table S3 tub "111". We then selected only those titled "conserved protein, unknown function" which result in the 42 genes found in Table S3 tub "42".
