# Supplementary material for: Identification and Validation of Toxoplasma gondii Mitoribosomal Large Subunit Components
Source: Microorganisms. 2022 Apr 21;10(5):863. doi: 10.3390/microorganisms10050863 (PMC9145746; doi:10.3390/microorganisms10050863)
Supplement: Supplementary file 1 [file microorganisms-10-00863-s001.zip › Figures S1 and S2.pdf]

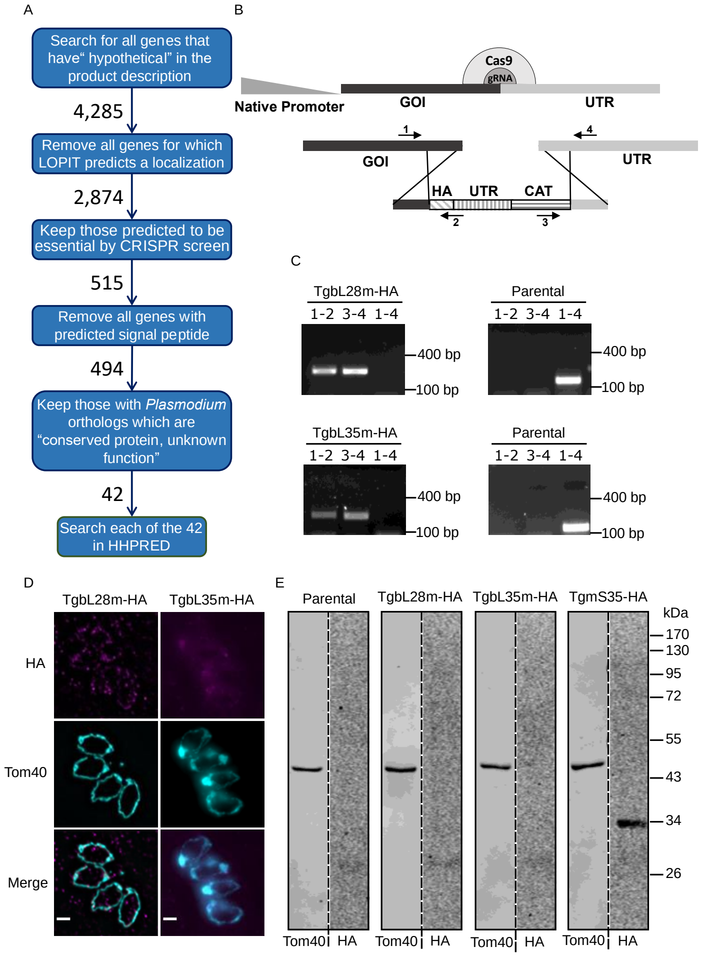

Figure S1 – Tagging of Tg*bL28m* and Tg*bL35m* results in undetected signal. (**A**) A scheme summarizing the *in silico* search steps taken to generate a focused list of genes used to search HHPRED for mitoribosom component homology (**B**) Scheme describing the genetic manipulation leading to endogenous tagging and showing the location of the primers used in (**C**). (**C**) Validation of the tag integration in the Tg*bL28m* and Tg*bL35m* loci via PCR analysis using primers 1, 2, 3, and 4 shown in (**B**). (**D**) Immunofluorescence micrographs taken with the two tagged lines probed with anti-HA antibody showing no detection of the HA tag. Tom40 has been used as mitochondrial marker. Scale bar = 1μm (**E**) Western blot analysis of total parasite lysate from each cell line, showing no signal detected for TgbL36m-HA and TgbL28m-HA, while TgmS35-HA, a previously validated mitoribosome component tagged with HA is detected. Each membrane was also probed with anti-Tom40 as loading control.

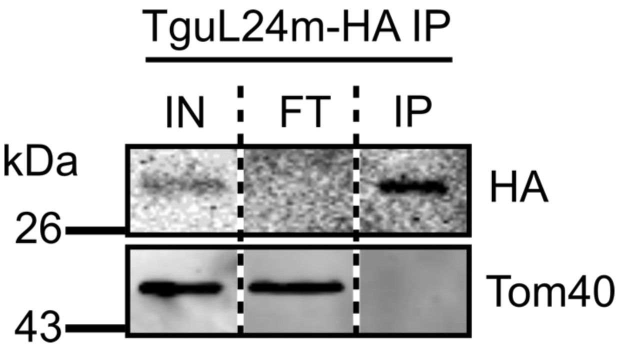

Figure S2 – Immunoprecipitation of TguL24m-HA. Western blot analysis of input (total parasites lysate, IN), flow through (FT) and elution (IP) fractions

showing the enrichment of HA-tagged TguL24m pulled down using anti-HA beads. The mitochondrial protein Tom40 is used as control showing it was fully lost in the flow through.
